# Protection of α-Tocopherol from UV-Induced Degradation by Encapsulation into Zein Nanoparticles

**DOI:** 10.3390/molecules29163911

**Published:** 2024-08-19

**Authors:** Sanghoon Kim

**Affiliations:** Plant Polymer Research Unit, National Center for Agricultural Utilization Research, Agricultural Research Service, USDA, 1815 N. University Street, Peoria, IL 61604, USA; sanghoon.kim@usda.gov; Tel.: +1-(309)-681-6260

**Keywords:** zein, tocopherol, nanoparticle, nanoencapsulation, UV degradation

## Abstract

Vitamin E is a fat-soluble vitamin with several forms. Among these, α-tocopherol (TOC) is preferentially absorbed and accumulated in humans. In the body, it acts as an antioxidant, helping to protect cells from the damage caused by free radicals. It is an organic chemical compound that undergoes degradation upon irradiation with UV light. To protect this bioactive chemical compound from UV light degradation, encapsulation was carried out using zein as a shell material. Due to the unique phase diagram of TOC in aqueous ethanol, the encapsulation efficiency was >99%. The size of encapsulated particles was ~300 nm or smaller, and the thickness of the shell wall was ~30 nm. The presented procedure offers the most simple and efficient encapsulation process that yields edible products. The investigation of the irradiation effect of UV on TOC revealed that the encapsulation effectively blocks UV light and prevents TOC from being degraded. The presented procedure offers an instantaneous and highly efficient encapsulation process, which yields edible products.

## 1. Introduction

Vitamin E is synthesized by plants and widely distributed in food sources, such as vegetable oils, seeds, nuts, avocados, peanut butter, margarine, fatty fish, and fish liver oil [1]. It consists of the four tocopherols and the four tocotrienols, where tocotrienols differ from tocopherols in that they have an unsaturated side chain. The four tocopherol isomers (i.e., α-, β-, γ-, and δ-tocopherol) are differentiated from each other by the number and position of the methyl group substitutions on the chromanol ring, as well as their bioactivity [2]. Tocopherols are free radical scavengers that oxidize tocopherols and tocotrienols to semi-stable radical intermediates. Unlike free radicals formed from PUFAs, the tocopheroxyl radical is relatively unreactive, thus stopping the destructive propagative cycle of lipid peroxidation [3]. Among the four tocopherol isomers, α-tocopherol (TOC) is the most active and the only isomer that is maintained in the plasma and recognized to meet human requirements [4]. TOC can reduce the risk of many chronic diseases, such as cancer, cardiovascular disease, and neurological disorders [3,5]. However, it is known that TOC is easily degraded by several environmental factors, such as UV light, oxygen, and temperature [6]. The susceptibility to these harsh conditions limits the availability of TOC in its bioactive form for health benefits.

To avoid these negative influences on TOC from the above-mentioned factors, various treatments have been attempted, such as complexation with b-cyclodextrins [7], inclusion into large-ring cyclodextrins [8], encapsulation in whey protein isolate/chitosan particles using oil-in-water emulsion [9], encapsulation in zein nanoparticles with gum arabic as an emulsifier [10], co-encapsulation with a natural polyphenol resveratrol in zein nanoparticles [11], etc.

Zein, a major storage protein in corn, is a prolamin. Therefore, it does not dissolve in water but in 70–90% aqueous ethanol. With this unique physical property and its amphiphilic character, zein has been used for the encapsulation of hydrophobic chemical compounds in the form of nanoparticles [12]. Since zein is also gastro-resistant, its application field has expanded to the fabrication of nanoparticles that deliver drugs [13].

The encapsulation process using zein begins with the preparation of a solution by dissolving both zein and the material to be encapsulated (i.e., core material) in 70–90% aqueous ethanol. Subsequently, water is added to lower the ethanol content of the prepared solution. Then, the phase-separated core material is spontaneously surrounded by zein to produce the encapsulation product [14,15]. Therefore, the dissolution of the core material in the same solvent that dissolves zein is a prerequisite for this process. The dissolved core material is supposed to be phase-separated upon mixing the solution with an antisolvent to form tiny emulsion particles. Since TOC is a lipophilic antioxidant, its solubility in water is very poor (only 1.9 × 10^−6^ mg L^−1^ at 25 °C) [6], dissolves only ~1.3% in 90% ethanol at 25 °C, and is less soluble in lower ethanol contents (Data is in this paper). This means that the conventional encapsulation process cannot be employed for the encapsulation of TOC into zein. Recently, this issue was resolved by incorporating an emulsifier into the zein–TOC–80% aqueous ethanol solution, stirring for an extended time, and rapidly injecting it into deionized water [10]. In another approach, zein solution in 80% aqueous ethanol was mixed with a TOC–absolute ethanol solution, added to an anti-solvent, followed by ethanol removal at elevated temperatures for an extended time [11]. In these procedures, processing times are too long to be used for mass production. In this report, a new, fast, and simple procedure for the encapsulation of TOC is introduced. The new encapsulation process does not require specialized equipment, and encapsulation is instantaneous.

A literature search indicates that there is no research on the influence of UV light on the degradation of TOC, except for the ones conducted decades ago. Pirisi et al. studied the photodegradation kinetics of TOC, and McVean, Kramer, and Liebler studied the inhibition effect of TOC on DNA photodamage [16,17,18]. This type of research does not provide any information on the relationship between the irradiated UV dose and the extent of degradation. In this report, therefore, the photodegradation of TOC by UV light was investigated using a chromatographic analysis method that yields quantitative data, and the obtained data were used for the evaluation of the encapsulation effect of TOC in nanocapsules made of zein.

## 2. Results and Discussion

### 2.1. Degradation of TOC by UV Light

**Figure 1:**
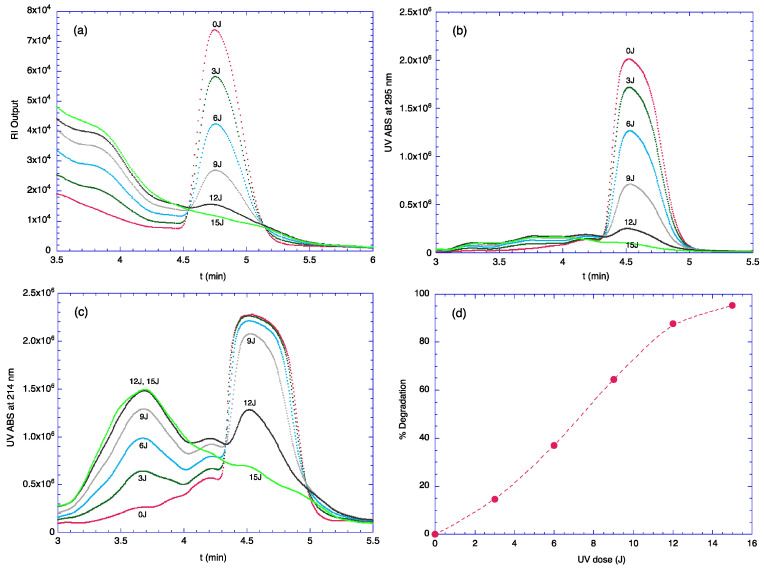
Degradation of TOC monitored with HPLC. (**a**) Degradation monitored with RI detector; (**b**) Degradation monitored at 295 nm; (**c**) Degradation monitored at 214 nm; (**d**) UV irradiation dose vs. degree of degradation. These data points were obtained from (**b**).

TOC molecules are degraded by the irradiation of UV light. According to the previous report, the absorption peak of TOC occurs at 294 nm [18]. Therefore, 306 nm UV lamps were employed for the investigation of the UV-induced degradation of TOC, as those lamps were commercially available. The UV-irradiated samples from various dose settings (from 0 to 15 Joules with 3 Joule increments) were analyzed using RP-HPLC. The irradiation effect is illustrated in Figure 1a,b by using RI and UV detectors, respectively. In the case of RI signals, the large peak (at ~3.1 min) from the eluent (i.e., methanol) was close, and the absorption intensity at 295 nm from the UV detector was used for the evaluation of the UV irradiation effect (Figure 1b). This graph clearly demonstrates the degradation of TOC by UV as a function of UV dose. The RI detector showed that the degraded TOC appears at ~3.9 min, and the UV detector (diode array detector) revealed that the UV absorption of this peak is maximized at ~214 nm. It should be noted that there is a 0.23 min time delay in the RI detector response. The chromatogram monitored at 214 nm is shown in Figure 1c. As the sample was irradiated with UV, a peak appeared at ~3.7 min and increased. Since this peak appeared earlier than that of TOC, the degradation product must be more polar than TOC. However, there is no evidence that the degradation products appear at ~3.7 min only. Therefore, the chromatogram obtained from the UV absorbance at 295 nm (Figure 1b) was used to investigate the relationship between UV dose and percent degradation (Figure 1d).

### 2.2. Conventional Encapsulation Process

The encapsulation process of a typical essential oil (i.e., menthol) into zein nanoparticles is explained in a previous paper [14]. The encapsulation process using zein as an encapsulant is known as the antisolvent precipitation method and can be briefly described as follows. The menthol solution prepared in 90% ethanol is phase-separated as the ethanol fraction of the solution is decreased by adding water to the solution. The phase-separated menthol forms emulsion droplets, and zein molecules surround them as soon as they are phase-separated from the solution. Thus, menthol-encapsulated particles are formed. This last process makes use of the amphiphilic character of zein molecules [19]. This encapsulation mechanism indicates that the material to be encapsulated needs to be soluble in the initial solution (i.e., 90% ethanol) and is phase-separated in the solution with lower ethanol content. The initial solution needs to be 90% ethanol, as zein dissolves in this solution, and the size of zein aggregates is minimal therein.

**Figure 2:**
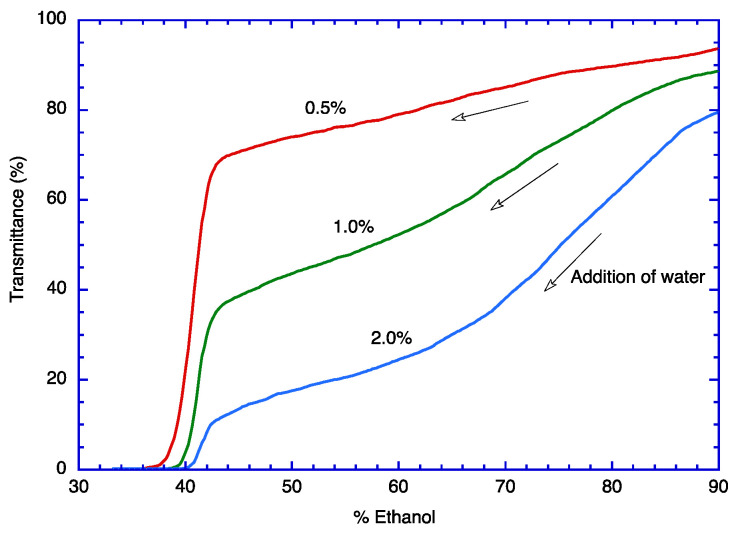
Aggregation of zein in 90% aqueous ethanol at lower content of ethanol; 0.5%, 1.0%, and 2.0% zein in 90% ethanol were titrated with water.

Figure 2 shows that zein forms aggregates in aqueous ethanol in <35% ethanol, although there is a slight variation in the turbidity development depending on the concentration of zein. As the aggregates formed from more highly concentrated zein will grow faster in the solvent medium, 2% zein developed turbidity a little earlier than 1% and 0.5% zeins. Figure 2 indicates that >35% ethanol is not adequate for the encapsulation of TOC with zein. Therefore, 30% ethanol was used for the encapsulation of TOC into zein (Table 1).

**Figure 3:**
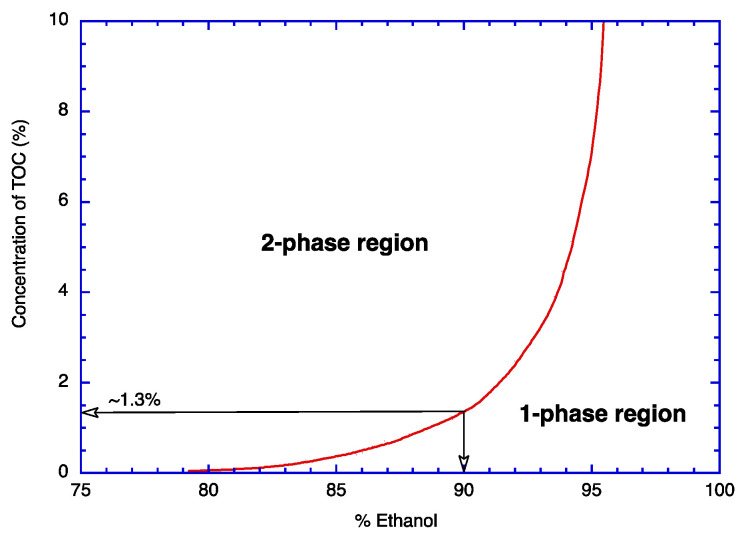
Phase diagram of TOC in aqueous ethanol solution.

According to the phase diagram of TOC (Figure 3), it is obvious that all the TOC molecules will be phase-separated in <75% ethanol solutions. In the case of many other chemical compounds (i.e., most essential oils), their solubility in aqueous ethanol gradually decreases as the content of ethanol decreases. Therefore, it is necessary to lower the ethanol content of aqueous ethanol as much as possible to maximize the quantity of phase-separated solutes. As an example, the case of menthol was shown in the previous paper [14]. In the case of TOC, however, its solubility in <75% ethanol is practically zero. This means that, unlike the case with menthol, all the TOC molecules can be encapsulated into zein without lowering the ethanol content of aqueous ethanol to 20%.

### 2.3. New Encapsulation Process for TOC

The phase diagram of TOC in aqueous ethanol (Figure 3) shows that the solubility of TOC in 90% ethanol is only ~1.3% at 25 °C. This situation is problematic, as the core material needs to be fully dissolved in 90% ethanol and phase-separates later as nonsolvent (i.e., water) is added to this solution. Therefore, the same process used for menthol cannot be employed for the encapsulation of TOC. To resolve this issue, the conventional procedure was modified. Instead of dissolving zein and the core material to be encapsulated in the same initial solution, three solutions were prepared: (1) zein in 90% aqueous ethanol, (2) TOC in 100% ethanol, and (3) water. Therefore, zein and TOC are fully soluble in each solution; however, upon mixing the solutions together, TOC is phase-separated. Compositions of the five sets of initial solutions used for this research are illustrated in Table 1. The developed three-solution mixing process has several advantages as follows: (1) this procedure is applicable to any core materials that are soluble in ethanol, (2) encapsulation is instantaneous, (3) encapsulation efficiency is very high, and (4) the encapsulation process is very simple.

### 2.4. TOC-to-Zein Ratio (TZR) and Shell Thickness

**Figure 4:**
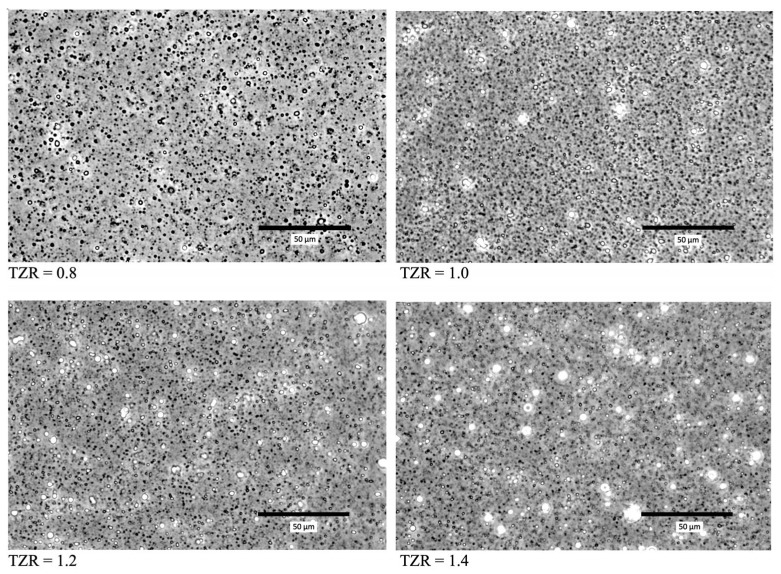
Micrographs of TOC/zein particles at different TZRs.

**Figure 5:**
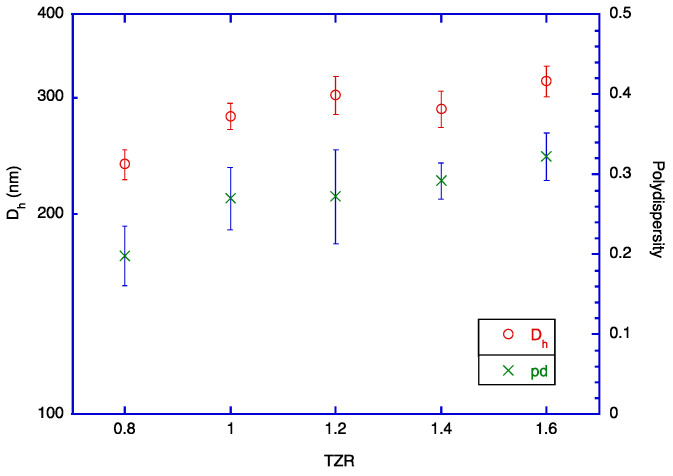
Hydrodynamic diameter of encapsulated particles at different TZRs. The concentration of zein was fixed at 1%, and the ethanol content of the final solution was 30%.

**Figure 6:**
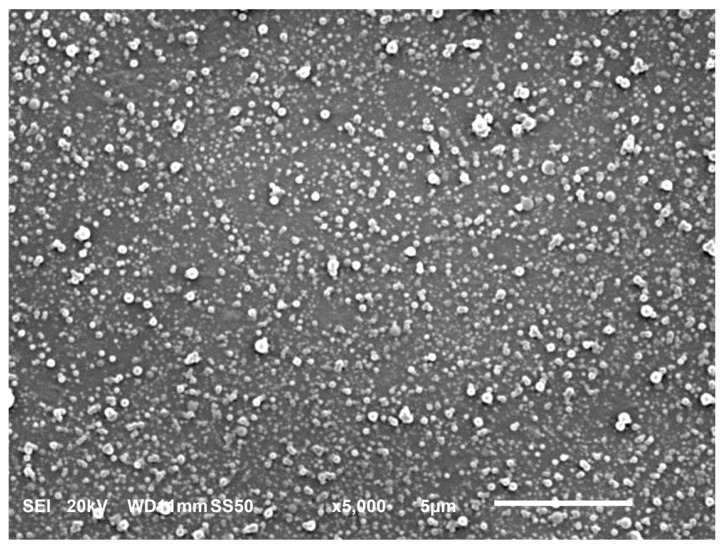
Scanning electron microscopic image of TOC–zein particles (TZR = 1.0).

The images of the prepared TOC/zein particles at four TZRs (TZR = 0.8, 1.0, 1.2, and 1.4) are shown in Figure 4. Overall, the average size of particles was very affected by TZR. However, the number of large particles increased as TZR increased. In the micrographs shown in Figure 4, large particles look white, but this is typical for images from phase-contrast microscopes. This is because the phase shifts from larger particles are larger than those from smaller particles. Therefore, the presence of larger-than-average particles could be noticed easily. As TZR gets larger, a larger amount of TOC is introduced into the solution. In this situation, the fraction of large particles increases at the same stirring condition. Therefore, the average particle size in the solution will increase. This viewpoint is supported by the particle size data obtained with DLS. The sizes of particles prepared from the five sets of solutions are shown in Figure 5 and listed in Table 2. For each set of solution mixtures, the amount of zein was the same, and the amount of TOC was varied to prepare the intended TOC-to-zein ratio (TZR = 0.8, 1.0, 1.2, 1.4, and 1.6). As expected, the polydispersity of particle sizes increased as TZR increased. The observation supports the aforementioned viewpoint. The SEM image of TOC-encapsulated zein particles is shown in Figure 6. With its much higher magnification factor than that of optical microscopic images, the shape of individual particles and polydispersity are more clearly shown. Since samples are heated while they are sputter coated and SEM images are taken under vacuum, the actual size of each particle is supposed to be slightly larger than it appears in the taken image. The dark spots at the center of some large particles are artefacts generated during the sputtering process.

In 30% ethanol, both TOC and zein are all phase separated, as shown in Figure 1 and Figure 2. This means the weight ratio of TOC and zein in each particle is the same as TZR. Since the weight of a component in each particle can be converted by dividing its weight by its density, the weight ratio of TOC and zein in each particle can be converted to the volume ratio (Table 2). As this volume ratio of TOC and zein is known, the volume fraction of the core can be calculated by the equation,

volume fraction of core = ((4/3) × π × r^3^)/((4/3) × π × R^3^) = (r/R)^3^ (1)

where R is the radius of the produced nanocapsule, and r is the radius of its core. Therefore, the radius of the core can be calculated from the equation,

r = (vol fraction of core)^1/3^ × R (2)

where R (i.e., half of the hydrodynamic diameter of the nanocapsule) can be obtained from the DLS experiment (Section 3.7). Thus, the shell thickness of each nanocapsule is calculated by subtracting r from R (Table 2). The calculation result shows that the shell thicknesses are in the range of 25–40 nm.

### 2.5. Encapsulation Efficiency (EE)

The phase diagram of TOC in aqueous ethanol showed that nearly all TOC molecules phase separated in <75% ethanol (Figure 3). These phase-separated TOC molecules will be encapsulated by zein if the surrounding medium is <35% ethanol (Figure 2). Since the encapsulation process is completed at 30% ethanol, the amount of TOC that is still in a dissolution state (i.e., the amount of unencapsulated TOC) is practically zero. Assuming that all the phase-separated TOC droplets are encapsulated by zein, EE is supposed to be close to 100% according to Equation (3) in Section 3.6. For the evaluation of this prediction, EE was experimentally determined using the procedure described in Section 3.6. The obtained experimental data revealed that EE was higher than 98% for all TZRs (Table 1). This result indicates that all the phase-separated TOC droplets are encapsulated by zein, and EE can be predicted from the phase diagram of the core material, TOC.

### 2.6. Encapsulation Effect on the Degradation of TOC

**Figure 7:**
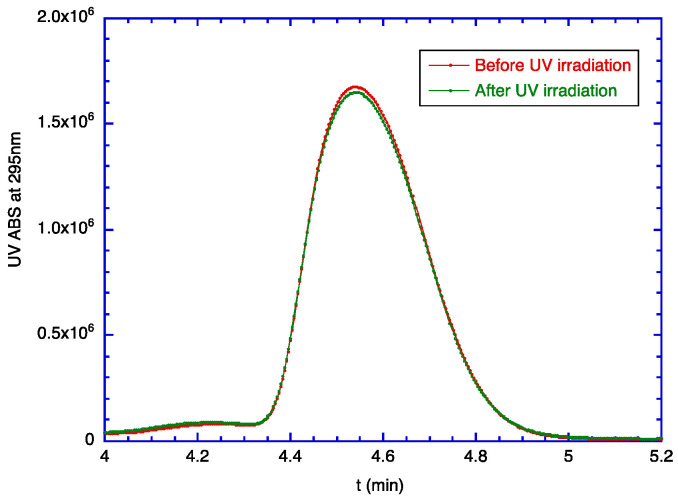
Encapsulation effect that suppresses degradation of TOC from UV irradiation. HPLC chromatograms of encapsulated TOC (TZR = 1.2) before and after the irradiation with 15 Joules of UVB. These HPLC chromatograms represent the average of three runs.

In Section 2.1, it was shown that TOC molecules were degraded by the irradiation of UV light. The encapsulation of zein was completed by surrounding the core material (i.e., TOC) with zein molecules. Therefore, TOC is not supposed to degrade if the passage of UV light is blocked by the shell material made of zein. Knowing that 15 Joules of UV light causes serious degradation of TOC (Figure 1b), the degradation of encapsulated TOC was evaluated by comparing it before and after the 15 Joules of UV irradiation. This evaluation was performed by comparing two encapsulated TOC samples, one with and the other without 15 Joules of UV irradiation. As the amount of intact TOC can be quantified by monitoring the absorption of 295 nm UV light (Section 2.1), both samples were dissolved in 90% ethanol to separate the TOC from the shell, and the intensities of peaks for intact TOC were compared. This experiment showed that (97.7 ± 1.4)% of TOC was unaffected by the irradiation of UV, indicating that the zein shell effectively shields TOC from UV irradiation (Figure 7). This shielding effect is explained as follows: the shell thickness of a zein nanocapsule is in the range of 20–45 nm (Table 2), while the diameter of an individual zein molecule is much less than 10 nm [19]. This means that the shell is composed of several layers of zein molecules. Therefore, it is concluded that these multi-layers of protein molecules can block the transmittance of UV light and effectively protect the core material from UV-induced degradation.

There are several recent reports that describe the encapsulation of vitamins into zein. In the case of water-soluble vitamins, it is very difficult to encapsulate them into zein, as zein does not dissolve in water. Therefore, encapsulation could be completed by using a custom-made confined impinging jet mixer to generate particles with a zein/chitosan double layer [20]. Chitosan had to be used to stabilize the produced zein particles because the surface of these particles was hydrophobic. Since zein has been used for the encapsulation of hydrophobic core materials during the last few decades, hydrophobic vitamins such as vitamins A, D, E, and K are expected to be easily encapsulated into zein. However, the solubility of these vitamins is very low in the solvent that dissolves zein. Therefore, the generation of tiny emulsion droplets by phase separation is not possible with conventional techniques. This issue could be resolved by using harsh mechanical breaking (homogenization) instead of inducing phase separation of core materials in the solution [21,22]. However, homogenizers generate large amounts of heat, heating up the solution. Vitamins are not stable compounds; for example, vitamin E (i.e., tocopherol) is very sensitive to temperature [6]. This might be the reason why research on the encapsulation of vitamins is rare. For commercialization, lower cost and easier scale-up are the most commonly considered factors in the food industry [23]. Compared with other techniques, the encapsulation process presented in this report is a much simpler (one-step process), faster (actual encapsulation time is <1 min), and more cost-effective process that can be readily scaled up.

## 3. Materials and Methods

### 3.1. Materials

Zein was purchased from Sigma-Aldrich (St. Louis, MO, USA) and defatted with n-hexane (Fisher Chemical, Fair Lawn, NJ, USA). For further purification, it was dissolved in 90% aqueous ethanol, filtered through a 2.7 μm syringe filter to remove aggregates, and the filtrate was freeze-dried. Both α-tocopherol (TOC) (Product #T3634, from vegetable oil, Type V, ~1000 IU/g) and methanol (HPLC grade) were obtained from Sigma-Aldrich. Ethanol was a product of Decon Laboratories, Inc. (King of Prussia, PA, USA).

### 3.2. Degradation of TOC by UV Light

The degradation of TOC by UV light was investigated by irradiating TOC solutions with 306 nm UV light (UVB) that was generated by a Model 234100 UV Crosslinker (Boekel Scientific, Feasterville-Trevose, PA, USA). Then, 4 mL of 0.5% TOC solution in pure ethanol was prepared in a quartz cuvette (internal dimension: 10 mm × 10 mm × 45 mm), and the dose setting was 3 Joules per irradiation. For the evaluation of UV-induced degradation on the encapsulated TOC, 100 mg each of the encapsulated TOC was prepared with and without 15 Joules of UVB irradiation in a quartz cuvette. After that, the TOC molecules encapsulated in zein particles were separated from zein, as described in Section 3.6, and the amount of intact TOC was evaluated by taking the RP-HPLC chromatogram with a UV detector set to 295 nm (Section 3.5).

### 3.3. Phase Diagram of TOC in Aqueous Ethanol by Transmittance Measurement

The phase diagram of the TOC/ethanol/water system was constructed by detecting the phase boundary with a custom-built turbidimeter. The turbidimeter was comprised of a 633 nm He–Ne laser, temperature-controlled sample block that holds a vial with 2.5 cm diameter, stirrer, and a laser power meter interfaced with a data logger [24]. During the transmittance measurement, the temperature of the sample block was set to 25 °C, and the sample solution was continuously stirred with a digitally controlled magnetic stirrer (>500 RPM). For the monitoring of the phase separation of TOC in aqueous ethanol, TOC solutions prepared in various ethanol contents were titrated with distilled water while each solution was stirred. During this process, the ethanol content in which the transmittance of the solution began to decrease was recorded as the phase boundary. The phase diagram for the TOC/ethanol/water system was constructed using these phase transition data obtained at 38 different ethanol/water compositions.

### 3.4. Preparation of TOC-Encapsulated Zein Particles

TOC was dissolved in absolute ethanol, and zein was dissolved in 90% ethanol. These two solutions were simultaneously poured into the water while the whole mixture was vigorously stirred (>500 RPM). The concentrations and volumes of TOC and zein were calculated to make the final concentration of zein and TOC ~1% and 0.8–1.6%, respectively. The details of the initial solutions are listed in Table 1.

### 3.5. Quantitative Analysis of TOC

Quantitative analysis of TOC was performed using reverse-phase high-performance liquid chromatography (RP-HPLC). To obtain RP-HPLC chromatograms, a Shimadzu LC-20 HPLC system (Shimadzu Corp., Kyoto, Japan) equipped with a refractive index detector (RID-10A) and a diode array detector (SPD-M30A) was used. The column used was a Luna 3 μm PFP(2) reverse-phase column (Phenomenex, Torrance, CA, USA), and the temperature for both the refractive index detector and the column was kept at 40 °C. The eluent was 100% methanol with a flow rate of 0.8 mL/min, and the injection volume of samples was 50 μL. This RP-HPLC procedure was used for the calculation of encapsulation efficiency of TOC in zein and for monitoring the degradation of TOC by UV irradiation.

### 3.6. Calculation of Encapsulation Efficiency (EE)

For the calculation of EE at various encapsulation conditions, the amount of unencapsulated TOC was measured by using RP-HPLC (Section 3.5). After the preparation of TOC-encapsulated particle suspension, the particles were precipitated by using the salting out technique [25]; 0.25 g of sodium chloride per 10 g particle suspension was added to each solution while stirring. The aggregated particles in each solution were separated by centrifuge at 3000× *g* for 10 min, and the amount of unencapsulated TOC in the supernatant was evaluated by the peak size for TOC in the chromatogram (UV detector set to 295 nm). Calibration was performed by taking the RP-HPLC chromatograms for pure TOC solutions with salt as well. For this analysis, 100% methanol was used as a mobile phase. EE was calculated by the equation,

(3) $$EE\left(\%\right)=\frac{total\;amount\;of\;TOC-amount\;of\;unencapsulated\;TOC}{total\;amount\;of\;TOC}\times 100$$

where the amount of unencapsulated TOC was measured by the quantitative analysis of TOC remaining in the supernatant after the salting-out process.

### 3.7. Hydrodynamic Diameter of Particles

The size of particles in each prepared suspension was measured with a NanoBrook Omni dynamic light scattering (DLS) instrument (Brookhaven Instruments Corp., Holtsville, NY, USA). Experiments were carried out at 23.0 °C. For each sample, measurements were made ten times, and the duration of each run was 10 s. Data from five sets of measurements were averaged for the analysis.

### 3.8. Optical Microscopy of TOC-Encapsulated Particles

The optical images of TOC-encapsulated particles were examined with an optical microscope (Model Axiovert 100, Zeiss, Waltham, MA, USA) with a phase-contrast objective (LD A-Plan, 20×) in conjunction with image analysis software. Microscopic images were captured with a digital camera (Model MU2003-BI, Amscope, Irvine, CA, USA), and the obtained images were processed with Preview (Version 11.0, Apple Inc., Cupertino, CA, USA) for the adjustment of brightness/contrast.

### 3.9. Scanning Electron Microscopy (SEM) of TOC-Encapsulated Particles

The SEM images of TOC-encapsulated particles were obtained using a JEOL JSM-6010LA Analytical Scanning Electron Microscope (JEOL USA Inc., Peabody, MA, USA). Samples were prepared following the procedure specified in Section 3.4 and diluted ten times with 30% aqueous ethanol. Then, 0.5 μL of solution was deposited on the surface of a 3 mm square silicon wafer attached to a piece of double-sided carbon tape, which was affixed to a 9.5 mm diameter JEOL aluminum SEM specimen mount (Ted Pella Inc., Redding, CA, USA). The solution was allowed to dry; then, each sample stub was coated using an SPI-Module gold sputter coater (Structure Probe, Inc., West Chester, PA, USA) and examined with SEM. 

### 3.10. Statistical Analysis

The statistical analysis was performed using KaleidaGraph (version 5.01, Synergy Software, Reading, PA, USA). Raw data from DLS measurements were processed using the software supplied by the manufacturer (9kpsdw, v.5.31) to obtain the mean hydrodynamic diameter and error bars via a multimodal analysis. Error bars for the graphs are calculated for a 95% confidence interval. Data were analyzed with ANOVA and Tukey–Kramer honestly significant difference test at *p* < 0.05 using JMP 15 program (SAS Institute, Cary, NC, USA).

## 4. Conclusions

In this research, it is shown that unprotected TOC is easily degraded by exposure to UV light. Examination of the degraded TOC with HPLC revealed that UV irradiation converts TOC to a highly polar compound, and the amount of degraded TOC is proportional to UV dose (i.e., exposure time to UVB). This research also demonstrated that the encapsulation of TOC into zein nanocapsules is an excellent way of protecting TOC from UV-induced degradation. A newly developed procedure for the encapsulation of TOC was proven to be successful for the purpose. In principle, any chemical ingredients that are soluble in ethanol and phase separate by the addition of water can be encapsulated into zein by using this procedure. In the case of encapsulation of TOC, an unusually high EE (>99%) could be achieved because its solubility in the final solution was close to zero. This encapsulation procedure produced nanocapsules with ~30 nm thick shell, and this shell was able to effectively block the UV light. This result indicates that UV-sensitive food ingredients can be protected from UV light by encapsulating them into zein. In short, this research presents a very simple process for the production of encapsulated TOC that is edible, and the encapsulated TOC is stable against degradation by UV light.

## Figures and Tables

**Table 1:** The composition of the initial solutions with different TOC-to-zein ratios.

Sample #	Solution A	Solution B	Solution C	Ethanol Content of the Mixture (%)	TZR *	D_h_ (nm) **	Polydispersity	EE (%)
A1	TOC 0.408 g + ethanol 6.0 g	zein 0.51 g + 90% ethanol 10.0 g	Water 34.0 g	30	0.8	238 ± 6 ^d^	0.20 ± 0.04 ^b^	98.6 ± 0.2 ^c^
A2	TOC 0.51 g + ethanol 6.0 g	zein 0.51 g + 90% ethanol 10.0 g	Water 34.0 g	30	1.0	281 ± 10 ^c^	0.27 ± 0.06 ^ab^	98.6 ± 0.2 ^c^
A3	TOC 0.612 g + ethanol 6.0 g	zein 0.51 g + 90% ethanol 10.0 g	Water 34.0 g	30	1.2	303 ± 6 ^b^	0.27 ± 0.04 ^ab^	98.7 ± 0.2 ^bc^
A4	TOC 0.714 g + ethanol 6.0 g	zein 0.51 g + 90% ethanol 10.0 g	Water 34.0 g	30	1.4	288 ± 6 ^bc^	0.29 ± 0.02 ^a^	99.2 ± 0.2 ^a^
A5	TOC 0.816 g + ethanol 6.0 g	zein 0.51 g + 90% ethanol 10.0 g	Water 34.0 g	30	1.6	318 ± 9 ^a^	0.32 ± 0.03 ^a^	98.8 ± 0.2 ^b^

* TOC-to-zein ratio; ** hydrodynamic diameter; ^a–d^ Means not sharing the same letter(s) are significantly different by Tukey-Kramer HSD test (*p* < 0.05) within a column.

**Table 2:** The physical dimensions of particles prepared from the five sets of solutions.

Sample #	TZR	Wt. Ratio (Core/ Shell)	Volume Ratio ^a^ (Core/ Shell)	Volume Fraction of Core ^b^	R ^c^ (nm)	r ^d^ (nm)	Shell Thickness ^e^ (nm)
A1	0.80	0.80	0.620	0.3827	119.0 ± 3.0	86.4 ± 2.2	32.6 ± 5.2
A2	1.0	1.0	0.775	0.4366	140.5 ± 5.0	106.6 ± 3.8	33.9 ± 8.8
A3	1.2	1.2	0.930	0.4819	151.5 ± 3.0	118.8 ± 2.4	32.7 ± 5.4
A4	1.4	1.4	1.085	0.5204	144.0 ± 3.0	115.8 ± 2.4	28.2 ± 5.4
A5	1.6	1.6	1.240	0.5536	159.0 ± 4.5	130.6 ± 3.7	28.5 ± 8.2

^a,b^ density of zein = 1.226; density of TOC = 0.950 at 20 °C. ^c^ R = radius of particle = D_h_/2. ^d^ r = radius of core, volume fraction of core = ((4/3) πr^3^)/((4/3) πR^3^) = (r/R)^3^, r = (volume fraction of core)^1/3^ × R. ^e^ shell thickness = R − r.

## Data Availability

The raw data supporting the conclusions of this article will be made available by the authors upon request.
